# STW 5 is effective in dextran sulfate sodium-induced colitis in rats

**DOI:** 10.1007/s00384-012-1473-z

**Published:** 2012-05-06

**Authors:** Walaa Wadie, Heba Abdel-Aziz, Hala F. Zaki, Olaf Kelber, Dieter Weiser, Mohamed T. Khayyal

**Affiliations:** 1 Department of Pharmacology, Faculty of Pharmacy, Cairo University, Cairo, Egypt; 2 Department of Pharmacology, Institute of Pharmaceutical Chemistry, University of Münster, Münster, Germany; 3 Scientific Department, Steigerwald Arzneimittelwerk GmbH, Darmstadt, Germany

**Keywords:** DSS colitis, Colonic responsiveness, STW 5, Cytokines

## Abstract

**Purpose:**

An herbal preparation, STW 5, used clinically in functional dyspepsia and irritable bowel syndrome, has been shown to possess properties that may render it useful in inflammatory bowel disease (IBD). The present work was conducted to study its effectiveness in a rat model of IBD.

**Methods:**

An experimental model reflecting ulcerative colitis in man was adopted, whereby colitis was induced in Wistar rats by feeding them 5 % dextran sulfate sodium (DSS) in drinking water for one week. STW 5 and sulfasalazine (as a reference standard) were administered orally daily for 1 week before colitis induction and continued during DSS feeding. The animals were then sacrificed, and the severity of colitis was evaluated macroscopically and microscopically. Colon samples were homogenized for determination of reduced glutathione, tumor necrosis factor-α, and cytokine-induced neutrophil chemoattractant-3 as well as myeloperoxidase, glutathione peroxidase, and superoxide dismutase. In addition, colon segments were suspended in an organ bath to test their reactivity towards carbachol, KCl, and trypsin.

**Results:**

STW 5 and sulfasalazine were both effective in preventing the shortening of colon length and the increase in both colon mass index and total histology score as well as the changes in biochemical parameters measured except changes in dismutase activity. DSS-induced colitis led to marked depression in colonic responsiveness to the agents tested ex vivo, an effect which was normalized by both drugs.

**Conclusions:**

The findings point to a potential usefulness of STW 5 in the clinical setting of ulcerative colitis.

## Introduction

Inflammatory bowel disease (IBD) in man is usually manifested either as ulcerative colitis (UC) or as Crohn's disease. In both conditions, the inflammatory processes in the gut are associated with disturbances in gastrointestinal motility [1, 2]. One animal model of UC involves feeding dextran sulfate sodium (DSS) in drinking water to rodents [3, 4]. The model is widely used for screening purposes because of its ease of induction, reproducibility, well-characterized mucosal injury [3], and its responsiveness to effective agents such as sulfasalazine. The aims of management of IBD are the induction and maintenance of remission. However, no maintenance medication has been shown to be completely effective for preventing relapse. The search, therefore, continues in the hope of finding effective and relatively non-toxic agents that could be useful in prolonging remission intervals while minimizing adverse reactions.

STW 5 consists of a fixed dose combination of well-characterized and standardized nine herbal extracts, namely bitter candytuft (*Iberis amara*), melissa leaf (*Melissa officinalis*), chamomile flower (*Matricaria recutita*), caraway fruit (*Carum carvi*), peppermint leaf (*Mentha piperita*), Angelica root (*Angelica archangelica*), milk thistle (*Silybum marianum*), greater celandine herb (*Chelidonium majus*) and liquorice root (*Glycyrrhiza glabra*) that has proved to be effective against functional gastrointestinal disorders such as functional dyspepsia and irritable bowel syndrome [5–7]. Khayyal et al. [8, 9] reported on its anti-ulcerogenic and mucosal protective effects and related at least part of its usefulness to its anti-inflammatory properties, a fact that prompted us to further explore the potential benefits of the drug in the DSS model of IBD.

## Materials and methods

### Drugs

STW 5 was provided in the form of its commercial preparation (Iberogast^®^) by Steigerwald Arzneimittelwerk GmbH, Darmstadt, Germany. The preparation is provided as a tincture containing 31 % alcohol. Sulfasalazine was obtained from El-Kahira Pharmaceutical Company, Cairo, Egypt and was used as a suspension in 1 % methylcellulose. The concentration of all drugs was adjusted so that the required dose per 200 g of rat was found in 1 mL of solution/suspension.

### Animals

Adult male Wistar rats, weighing 150–200 g each, were obtained from the Modern Veterinary Office for Laboratory Animals, Cairo, Egypt and were left to acclimatize for 1 week before subjecting them to experimentation. They were provided with a standard pellet diet and were given water ad libitum. The animals were kept at a temperature of 22 ± 3°C and a 12-h light/dark cycle as well as a constant relative humidity throughout the experimental period. The study was approved by the Ethical Committee for Animal Experimentation at the Faculty of Pharmacy, Cairo University, Cairo, Egypt.

### Induction of colitis

Colitis was induced by adding 5 % DSS (molecular weight of 37–40 kD; TdB Consultancy, Uppsala, Sweden) to the drinking water, ad libitum for 7 days [3, 4].

### Experimental design

Two sets of experiments were carried out: one with a view to determine changes in histological and biochemical parameters and one to study motility changes ex vivo.

#### Study of effect of STW 5 on histological and biochemical parameters

Rats were randomly assigned to seven groups of ten animals each as follows: two control groups given 31 % ethanol (vehicle of STW 5), four STW 5-treated groups (0.5, 1, 2, and 5 mL/kg), and one sulfasalazine-treated group (300 mg/kg). The drugs were administered orally once per day for 1 week while keeping the animals on a normal pellet diet and water ad libitum. DSS (5 %) was then added to the drinking water of all groups except one of the control groups (considered as normal control, not receiving DSS). DSS treatment was continued for 1 week while keeping the rats under the same drug regimen during that time. Animals were weighed just before DSS treatment and just before autopsy to determine whether colitis had an effect on body weight.

Twenty-four hours after the last dose of treatment, the rats were sacrificed by cervical dislocation. The colon was excised and its length measured. The colon was opened longitudinally, rinsed in ice-cold normal saline, cleaned of fat and mesentery, blotted on filter paper, and weighed. The ratio of colon weight in milligrams to total body weight in grams was taken as the colon mass index and was used as a measure of the degree of colonic edema and severity of inflammation. The colon was then cut longitudinally into two parts. One specimen was fixed in 10 % formalin and preserved for histological examination, and the other was homogenized in ice-cold normal saline to obtain a 10 % homogenate for assessment of the chosen biochemical parameters.

##### Determination of biochemical parameters

The colon homogenate was divided into four aliquots for determining the following parameters:

##### Reduced glutathione

One aliquot was deproteinized with ice-cold 5 % sulfosalicylic acid then centrifuged at 3,000 rpm for 20 min. The supernatant was used for spectrophotometric estimation of glutathione (GSH) [10].

##### Myeloperoxidase activity

An aliquot was mixed with an equal volume of 100 mmol/L phosphate buffer pH 6 containing 1 % hexadecyltrimethylammonium bromide. The mixture was freeze-thawed, sonicated for 10 s and centrifuged at 10,000 rpm for 15 min at 4°C. The supernatant was used for spectrophotometric estimation of myeloperoxidase (MPO) activity [11].

##### Tumor necrosis factor alpha and cytokine-induced neutrophil chemoattractant-3

An aliquot was centrifuged at 13 000 rpm for 30 minutes at 4°C. The supernatant was used for assaying tumor necrosis factor (TNF)-α and cytokine-induced neutrophil chemoattractant (CINC)-3 using specific enzyme-linked immunosorbent assay kits (R&D Systems GmbH, Wiesbaden, Germany).

##### Superoxide dismutase and glutathione peroxidase

An aliquot was ultra-centrifuged at 105 000 g for 15 minutes at 4°C. The supernatant was used for the assay of superoxide dismutase (SOD) and glutathione peroxidase (GPx) using specific spectrophotometric kits (Biodiagnostic, Giza, Egypt).

##### Histopathological examination

Transverse sections, 4-6 μm in size, were prepared from paraffin-embedded colon segments from each animal of all experimental groups except those treated with the two lower doses of STW 5. The sections were stained with hematoxylin and eosin (H&E) and examined under a light microscope. They were graded individually by a pathologist blinded to the treatment regimen. Each section was assigned a damage score between 0 and 3 for each of five parameters, namely, necrosis of epithelium, inflammatory infiltrate in lamina propria, inflammatory infiltrate in submucosa, dilatation of crypts, and submucosal edema.

The scores for the five parameters measured for each rat were summed to obtain the “total histology score,” being maximally 15 (three being the maximum for the five parameters examined). The data were then represented using a box plot.

#### Colonic responsiveness ex vivo

Rats were allocated to four groups of animals, each comprising six rats: two control groups, one group treated with sulfasalazine (300 mg/kg) and one group with STW 5 (5 mL/kg). They were subjected to the same treatment procedure as above.

After sacrifice, segments of the colon approximately 20 mm in length were taken 3 cm from the anal orifice and suspended longitudinally under a 1-g load in a 30-mL water jacketed organ bath containing Krebs-Henseleit solution of the following composition (millimole per liter): NaCl, 119.0; KCl, 4.7; CaCl_2_, 2.5; MgSO_4_, 1.2; KH_2_PO_4_, 1.2; NaHCO_3_, 25.0; glucose, 5.5. The bath was maintained at 37°C and aerated with carbogen.

The preparation was allowed to equilibrate for 1 h. Isotonic contractions were recorded for carbachol (0.03–1 μmol/L) and KCl (9.09–72.7 mmol/L). The relaxant effect of a protease-activated receptor (PAR)-2 agonist, trypsin (MP Biomedicals Inc., Solon, USA) was evaluated on a muscle strip pre-contracted with a submaximal dose of carbachol. Trypsin (30 μmol/L) was applied 5 min after the addition of carbachol and the extent of relaxation measured.

### Statistical analysis

SPSS 17.0 was used for data management and data analysis. Mean and standard error described continuous variables with median and range for discrete data. Parametric and non-parametric ANOVA compared means and medians of different study groups. When significant, pair-wise comparisons were done using most restrictive tests: Scheffe test. *P* value was considered significant at 0.05 or less.

## Results

### Body weight

**Fig 1:**
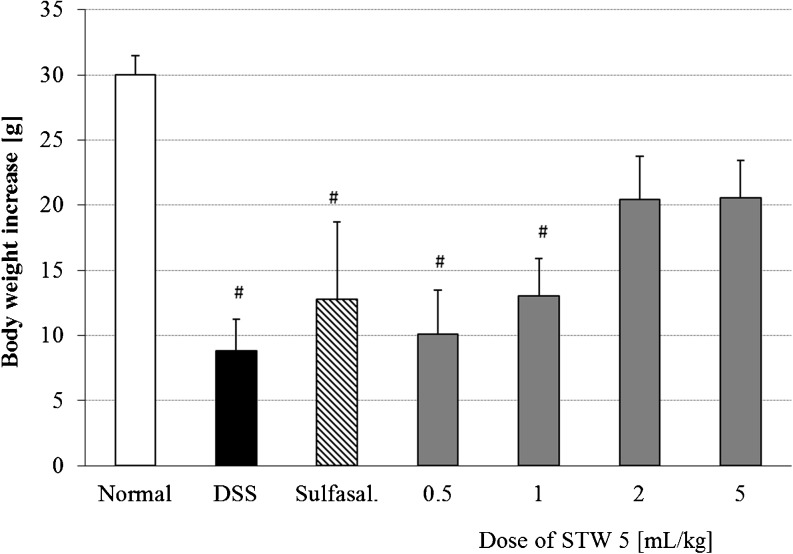
Effect of pretreatment with STW 5 on the increase in body weight of animals with DSS-induced colitis measured from the time of induction of colitis until sacrifice. Data are expressed as means ± SEM of at least eight animals. ^#^ *P* ≤ 0.05 vs. normal control

DSS-induced colitis led to a dramatic inhibition in the rate of weight gain of rats by ca. 70 % (*p* < 0.001). Pretreatment with STW 5 (2 and 5 mL/kg) tended to restore the normal rate of weight gain (*p* = 0.09) while the lower doses of STW 5 and sulfasalazine (300 mg/kg) had little effect (Fig. 1).

### Colon length and colon mass index

**Fig. 2:**
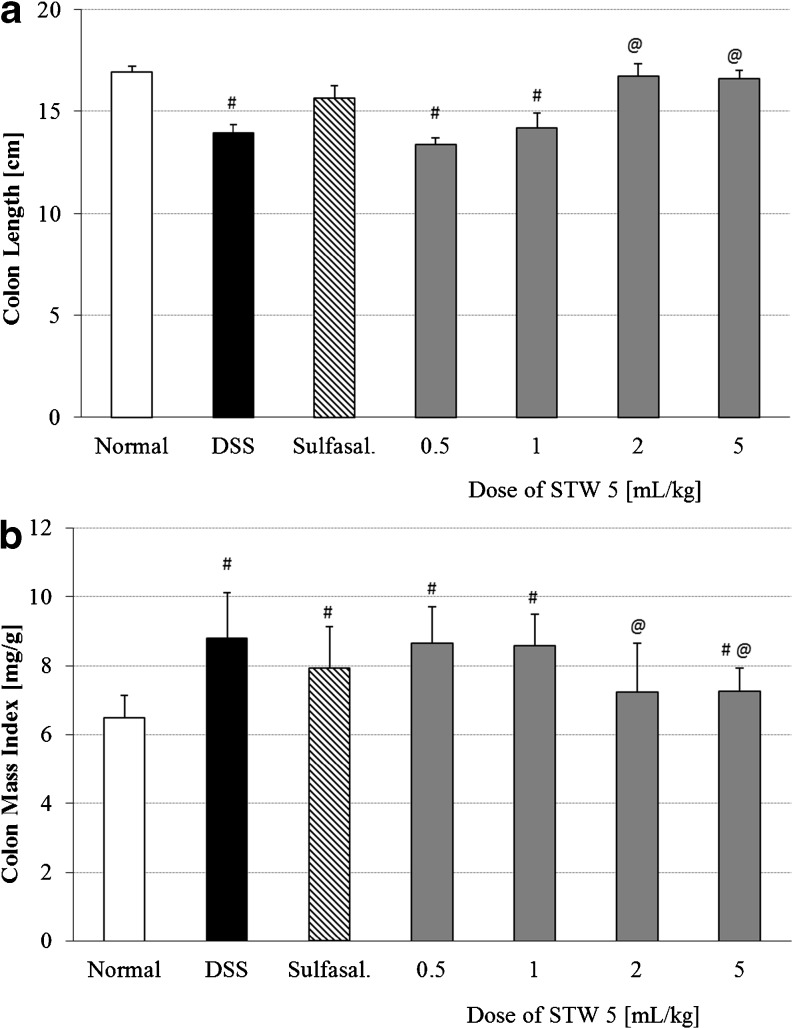
Effect of STW 5 on colon length **(a)** and colon mass index **(b)** in rats with DSS-induced colitis. Data are expressed as means ± SEM of at least eight animals. ^#^ *P* ≤ 0.05 vs. normal control, ^@^ *P* ≤ 0.05 vs. DSS control

Induction of colitis led to a reduction in colon length by 17 % as compared to the normal control group (Fig. 2a). This was associated with a 35 % increase in colon mass index (Fig. 2b). Pretreatment with STW 5 tended to prevent these changes in a dose-dependent manner. The effect of the two higher doses of STW 5 was comparable to that of sulfasalazine (Fig. 2; *p* < 0.001 and *p* = 0.002 for colon length and colon mass index respectively).

### Biochemical parameters

**Fig. 3:**
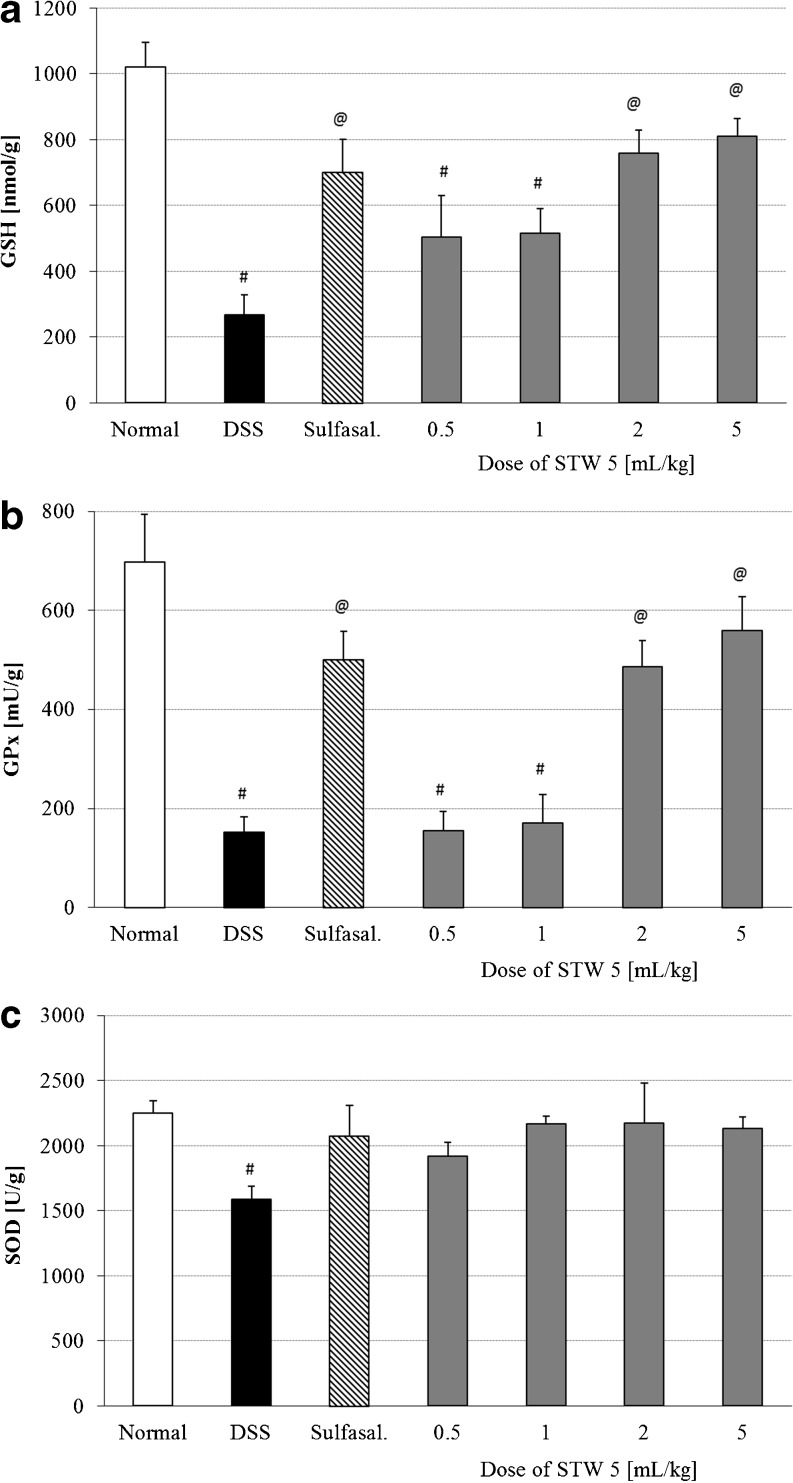
Effect of STW 5 on reduced glutathione (GSH) levels (**a**), glutathione peroxidase (GPx) activity (**b**) and superoxide dismutase (SOD) activity (**c**) in colonic tissue of rats with DSS-induced colitis. Data are expressed as means ± SEM of at least eight animals. ^#^ *P* ≤ 0.05 vs. normal control, ^@^ *P* ≤ 0.05 vs. DSS control

The induction of colitis was associated with changes in the various parameters indicative of oxidative stress. This was evidenced by a marked reduction in colonic content of GSH (Fig. 3a) as well as the activities of GPx (Fig. 3b) and SOD (Fig. 3c). Pretreatment with STW 5 dose-dependently reversed the deranged levels of GSH and GPx (Fig. 3a and b, respectively; *p* < 0.001). The reduction in colonic SOD activity induced by DSS was largely prevented by pretreatment with either STW 5 or sulfasalazine (Fig. 3c).

**Fig. 4:**
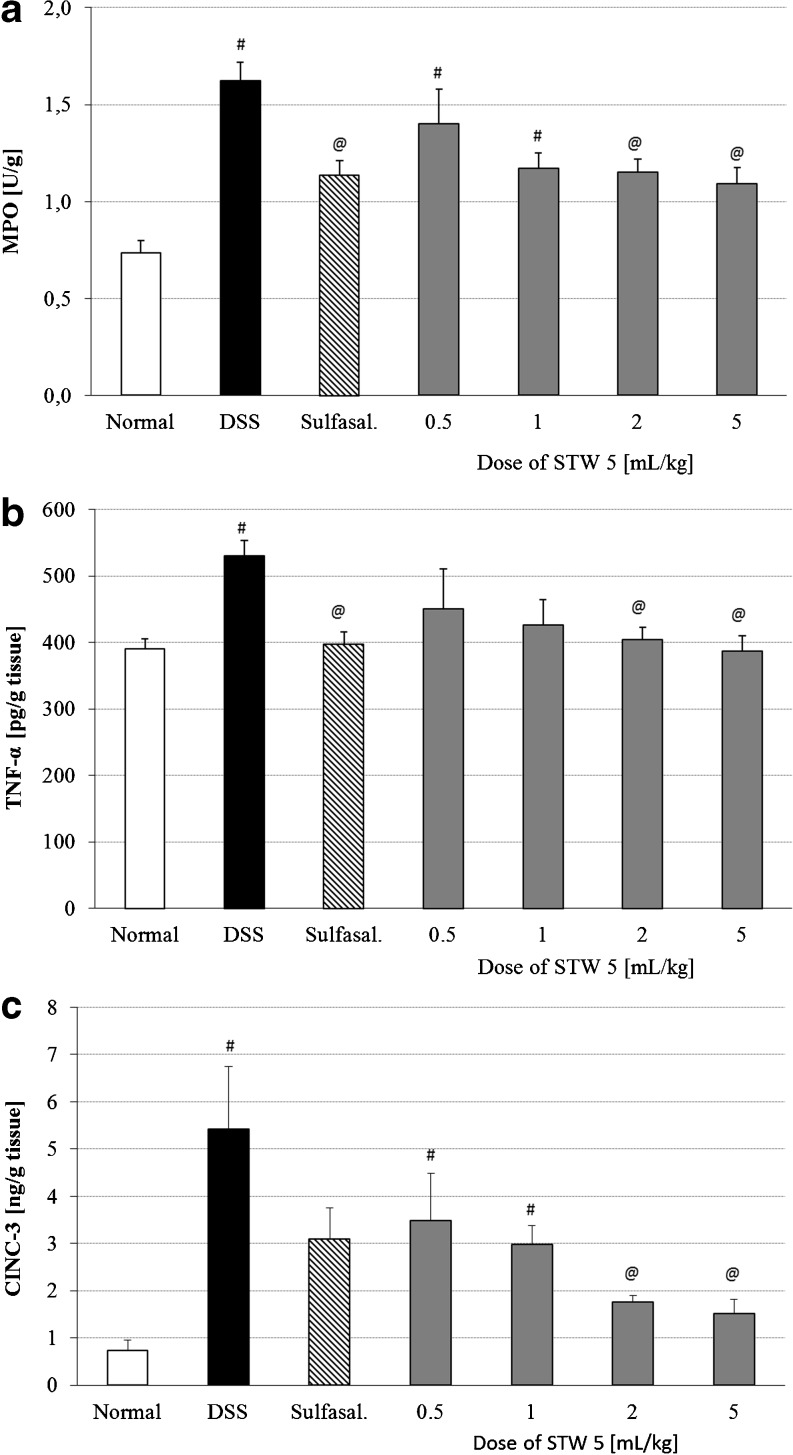
Effect of STW 5 on inflammatory parameters in colonic tissue of rats with DSS-induced colitis. **a** Myeloperoxidase (*MPO*) activity, **b** TNF-α levels, **c** CINC-3 levels. Data are expressed as means ± SEM of at least eight animals. ^#^ *P* ≤ 0.05 vs. normal control, ^@^ *P* ≤ 0.05 vs. DSS control

DSS-induced colitis led to a significant increase in MPO activity in the colon, an effect which was largely prevented by pretreatment with either STW 5 or sulfasalazine (Fig. 4a; *p* < 0.001).

The inflammatory mediators, TNF-α and CINC-3 (Fig. 4b and c, respectively), were elevated in colonic tissue of DSS treated rats, but the rise was prevented by pretreatment with either STW 5 or sulfasalazine (*p* < 0.001).

### Histopathological examination

**Fig. 5:**
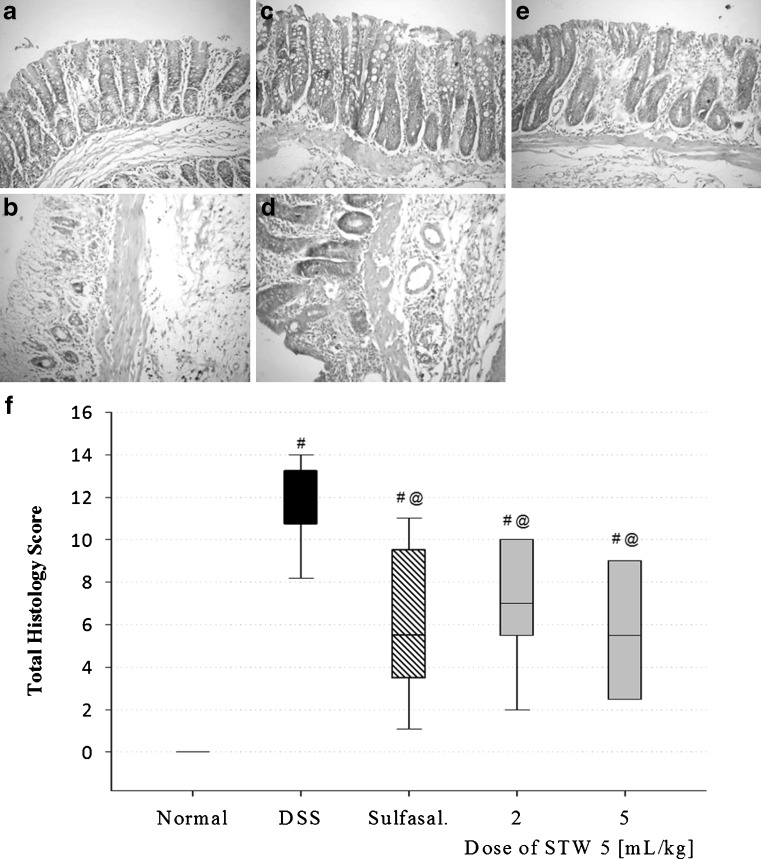
Effect of STW 5 on histopathological changes of rat colon in DSS model of colitis. **a** Normal control rat: normal histological structure of mucosa, **b** DSS control rat showing necrosis of epithelium, distortion of crypts, inflammatory infiltrate in lamina propria and submucosa as well as submucosal oedema, **c** sulfasalazine pre-treated rat showing nearly normal structure, **d** STW 5 (2 mL/kg) pretreated rat showing moderate inflammatory infiltrate in lamina propria and submucosa, **e** rat pre-treated with STW 5 (5 mL/kg) showing minimal changes. (H&E staining, ×200 original magnification). **f** Total histology score, data are expressed as box plots of the median of at least eight animals. ^#^ *P* ≤ 0.05 vs. normal control, ^@^ *P* ≤ 0.05 vs. DSS control

The major elements of colonic inflammation include crypt destruction, mucosal ulceration, and infiltration of lymphocytes into the mucosal tissue. Representative histological images of H&E-stained colon sections from each group are shown in Fig. 5. In contrast to normal control animals (Fig. 5a), DSS-treated animals (Fig. 5b) showed marked necrosis of the epithelium, intraluminal accumulation of mucous exudates, dilatation of the crypts, and submucosal edema. These changes were associated with massive inflammatory cell infiltration in the lamina propria and submucosa. The infiltrated inflammatory cells included polymorphonuclear leucocytes, lymphocytes, and plasma cells.

The total histology score was markedly increased in DSS-treated rats (Fig. 5f). Pretreatment with STW 5 largely protected against the histological changes induced by DSS as evidenced by the lesser severity of the above parameters. The inflammatory infiltration in the lamina propria and submucosa was only mild to moderate (Fig. 5d, e), and the total histology score was markedly decreased (Fig. 5f). The higher the dose of the drug, the greater was its protective effect. The effect of STW 5 was similar to a large extent to the effects of sulfasalazine (Fig. 5c, f).

### Colonic motility

**Fig. 6:**
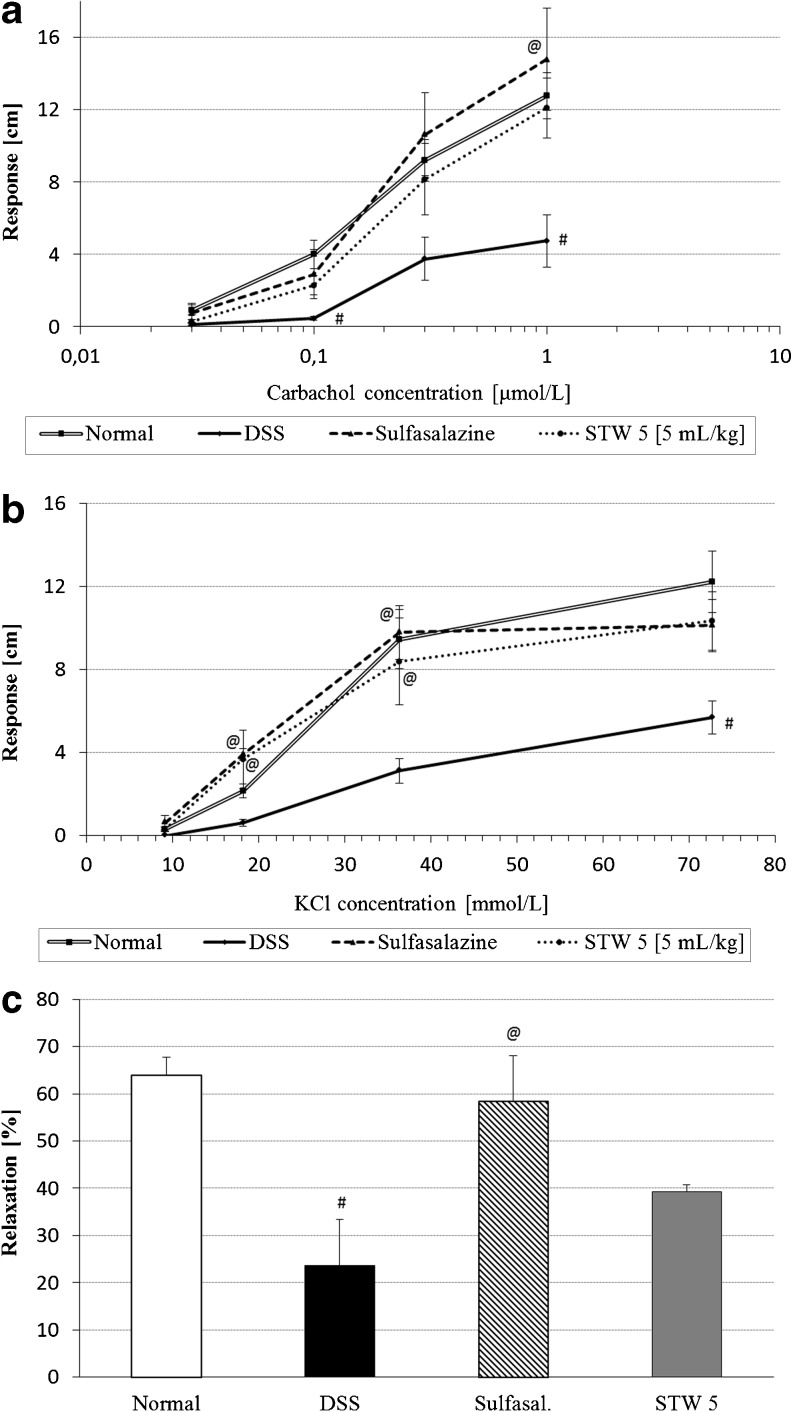
Effect of STW 5 on the colonic motility changes induced by DSS. **a** Non-cumulative concentration–response curves to carbachol, **b** non-cumulative concentration-response curves to KCl, **c** relaxant effect of trypsin after pre-contraction with carbachol [trypsin (30 μmol/L) was applied 5 min after addition of submaximal dose of carbachol]. Data are expressed as means ± SEM of at least four animals. ^#^ *P* ≤ 0.05 vs. normal control, ^@^ *P* ≤ 0.05 vs. DSS control

Colonic strips from rats with colitis showed depressed responsiveness to carbachol and KCl, an effect which tended to be prevented by both STW 5 and sulfasalazine (Fig. 6a, b, respectively).

Trypsin (30 μmol/L) was found to cause a 64 % relaxation of the carbachol-induced contraction of the normal rat colon. The extent of relaxation was markedly reduced in colons of rats with DSS-induced colitis, reaching only 24 %. Pretreatment with either sulfasalazine or STW 5 tended to restore the relaxing effect of trypsin to reach 58 % and 39 %, respectively (Fig. 6c).

## Discussion

The effect of STW 5 on various parameters that have been deranged as a result of inducing colitis through the administration of DSS in rats was studied. Macroscopically, DSS-induced colitis resulted in a marked reduction in the rate of growth of the animals. This feature of DSS colitis resembles the common problem of weight loss observed in IBD patients [12–14]. STW 5 in the higher dose range as well as sulfasalazine tended to restore the rate of weight gain. The effects on weight gain correlated well with the changes in colon length and colon mass index. The DSS-induced increase in colon mass index may be related to the submucosal edema shown histologically while the colon shortening may be associated with its thickening due to edema and infiltration of inflammatory cells into the lamina propria and submucosa [15, 16]. STW 5 led to marked reduction in the inflammatory infiltrate in both lamina propria and submucosa and dose-dependently protected against changes in colon length and mass index.

Neutrophil infiltration is usually associated with increased MPO activity, a parameter which correlates well with the severity of the lesions in acute DSS-induced colitis [17]. MPO itself possesses cytokine-like properties and can activate neutrophils [18] resulting in the release of a wide range of inflammatory mediators and reactive oxygen species. A decrease in MPO activity after pretreatment with STW 5 is therefore indicative of a reduction in neutrophil infiltration and reduction of the inflammatory process. Similar effects were recently reported in a model of reflux esophagitis [19].

The increased level of oxidative stress in the present study was associated with marked depletion of the cellular antioxidant, GSH as was reported in earlier studies [20, 21]. Other related parameters, such as the activities of the two antioxidant enzymes, SOD and GPx were also decreased. Such effects have also been reported in IBD patients [22]. Pretreatment with STW 5 protected dose dependently against the rise in free radical production by DSS, evidenced by restoration of colonic GSH, SOD, and GPx levels. Earlier authors reported that STW 5 possesses potent antioxidant activity in several in vitro models by virtue of its radical-scavenging activity and its ability to specifically inhibit metabolic pathways leading to cellular release of free radicals [23, 24].

Other factors that may be involved in the inflammatory process in human IBD as well as in DSS colitis include pro-inflammatory cytokines such as interleukin (IL)-1β and TNF-α [25–27]. Both TNF-α and IL-1β have been implicated in stimulating the production of IL-8 [28], a chemokine implicated in the pathophysiology of IBD [29]. One of the functional analogues of IL-8 in rodents is CINC-3, a potent chemotactic factor for neutrophils. The levels of TNF-α and CINC-3 were significantly raised in the colon following induction of colitis by DSS. Pretreatment with STW 5 dose dependently prevented the rise in both TNF-α and CINC-3. The reduction in TNF-α levels by STW 5, which was also described in several other models of GI inflammation [8, 9, 19, 30], may be explained partly by the previously reported inhibition of the translocation of nuclear factor-kappa B (NF-κB) [31]. NF-κB is believed to play a major role in the regulation of pro-inflammatory gene transcription [32].

Colitis is often associated with bowel motility changes leading to either constipation or diarrhea in patients with IBD [33, 34]. Colonic contractility has been reported to be deranged also in animal models of colitis [35] and to persist even after resolution of inflammation [36]. Both receptor-dependent and -independent mechanisms contribute to the altered smooth muscle function during colitis [37]. Colonic tissue from rats with DSS-induced colitis exhibited reduced contractility to both a receptor-dependent muscarinic agonist, carbachol, and a receptor-independent one, KCl, which stimulates smooth muscle directly by depolarizing the intact cell membranes.

The findings are suggestive of a reduced responsiveness of the colon to both parasympathetic stimulation and to direct muscle stimulation by depolarizing agents. Although the myogenic mechanism for decreased motility of inflamed intestinal tissue have not been well examined, some reports suggest that it involves a decreased activity of voltage-dependent L-type Ca^2+^ channels [38, 39], an effect that has been shown to be reversed by NF-κB inhibitors [39]. Since STW 5 was shown to inhibit the translocation and hence activation of NF-κB [31] the observed effects of the drug on restoring normal colonic responsiveness may be due to its anti-inflammatory effect, and hence conservation of L-type Ca^2+^ channel activity.

On parallel lines, Michael et al. [30] reported that in vitro induction of inflammation in the jejunum and small intestine by intraluminal instillation of TNBS led to a decreased response to acetylcholine, but the response was restored when STW 5 was instilled concurrently, thereby reducing histopathological inflammatory changes in the mucosa. Earlier studies had shown that STW 5 increased the tone of intestinal or colonic preparations, but had an antispasmodic action when tested against acetylcholine or histamine induced contractions [40], suggesting that STW 5 by virtue of one or more of its components may interact with the muscarinic and/or histaminergic receptors. Studies by Simmen et al. [41] in vitro showed that STW 5 indeed can bind to muscarinic M_3_ receptors in the intestine as well as to some serotonergic receptors. The modulatory effect of STW 5 may therefore be partially related to up- or downregulation of respective receptors.

As intestinal inflammation is associated with the generation and release of proteases that are potential activators of PAR-2, a receptor known to modulate intestinal motility, identifying the role of PAR-2 in the present model was of interest. Trypsin was found to inhibit carbachol-induced contractions of the normal rat colon. After colonic inflammation induced by DSS, the inhibitory effect of trypsin on carbachol-induced contractions was suppressed. However, pretreatment with STW 5 showed only a tendency to reduce the effect of trypsin.

In conclusion, the present findings show that STW 5 prevents both the colonic inflammatory changes and the motility disturbances in the DSS model of rat colitis. Restoration of normal responsiveness is probably related, at least in part, to the anti-inflammatory properties of the drug. If the present findings can be confirmed in a clinical setting, STW 5 may possibly have some therapeutic usefulness in the management of human IBD. It could be used as an add-on to standard medication to help maintain remission and ameliorate motility disturbances experienced by IBD patients.
